# Relative robustness of NOEC and ECx against large uncertainties in data

**DOI:** 10.1371/journal.pone.0206901

**Published:** 2018-11-28

**Authors:** Yoshinari Tanaka, Kensei Nakamura, Hiroyuki Yokomizo

**Affiliations:** Center for Health and Environmental Risk Research, National Institute for Environmental Studies, Tsukuba, Ibaraki, Japan; VIT University, INDIA

## Abstract

The relative ability of the NOEC (no-observed-effect concentration) and EC_*x*_ (the effect concentration corresponding to *x*-percent response) to determine benchmark toxicant concentrations, which are expected to ensure environmental safety, when there are large uncertainties in data was investigated with Monte Carlo simulations. We assumed a hypothetical true concentration-response function, and examined how random fluctuations of responses around the true responses affected the NOEC and EC_*x*_ values. For assessment of the relative performances of these endpoints, we adopted two criteria: how large uncertainties were allowed for the minimum requirement for safety to be met, and the probability with which the estimated endpoints exceeded the minimum requirement for safety. The results of simulations indicated that, when there were small uncertainties in the data, performance of the NOEC was comparable with or slightly better than the EC_*x*_ (EC_5_ and EC_10_) in providing benchmark concentrations that satisfied the minimum requirement for safety. With larger random variation of data (the coefficient of variation in responses between replicates within treatments or in the control was noticeably larger than 10 percent), the NOEC performed considerably worse than the EC_*x*_ in terms of the frequency of simulated runs in which the endpoints exceeded the minimum requirement of safety. We conclude that the NOEC is as relevant as the EC_*x*_ for risk assessment of chemicals under limited situations.

## Introduction

In the last two decades, the no-observed effect concentration (NOEC) has been severely criticized as a summary statistic in ecotoxicology [1,2,3]. As an alternative measure, the concentration that is predicted to induce a particular rate of response (*x*-percent effect concentration, EC_*x*_) or the model-based no-effect concentration (NEC) has been advocated [2,3]. The criticisms for the NOEC and related concepts are mainly directed towards the following two properties, which would be inappropriate for endpoints in toxicology. (1) A NOEC value is equated to one of the test concentrations and is largely dependent on the test design. (2) NOECs depend on the power of statistical hypothesis testing, which is based on pairwise comparisons between one of the exposure treatments and the control. The second property makes NOECs tend to be inversely associated with sample sizes and the number of test concentrations, and results in NOECs being positively associated with the magnitudes of random variations (uncertainties) of data. However, it should be noted that this downside of NOEC may be partly circumvented by applying more recent methods that take the concentration-response trend into the NOEC determination [4,5].

Hypothesis testing for statistically significant responses to toxicants with a particular concentration inevitably depends on the testing power, which is reduced by decreases in the sample size and increases in the random variations of the data. Thus, the NOEC cannot be in principle an unbiased estimate of the true value of the no-effect concentration, if it exists, and “rewards bad experiments” [3]. On the one hand, it is regarded in the standard statistics as one of the most relevant features of summary statistics to be unbiased as regard to expected values of the statistics not systematically increasing or decreasing in relation to sample sizes or the testing power [6,7,8]. In comparison to the NOEC, the EC_*x*_ might be less prone to the criticism as it is determined by two processes: the model-fitting process, in which the whole dataset is integrated into the model to derive the best estimates of model parameters, and the extrapolation process, in which unbiased estimates of the EC_*x*_ with an arbitrary response rate *x* are specified based on the best-fit model.

However, there are always uncertainties, to a greater or lesser degree, in toxicological data, and some types of uncertainties may not be entirely incorporated in the model-based estimation process [9,10], unless the confidence interval are reported. In particular, long-term chronic toxicity data tend to suffer severe limitations in numbers of test concentrations and replicates per concentration [5]. It is also a hard task to predict how the error distributions, which is a basic assumption of any model, influence the extent and the pattern of uncertainties in the estimated endpoints. Therefore, there is a danger that the NOEC and the EC_*x*_, even if it is specified at a small response rate, may be overestimated due to random errors of data to such an extent that it is higher than the marginal toxicant concentrations that exert real impacts on the environment. How often uncertainties of data cause an overestimate of the EC_*x*_ and the NOEC has not been rigorously investigated to provide any corroboration for the argument about the relative performance of the EC_*x*_ and the NOEC.

For purposes of risk assessment or regulation of chemicals, we have stronger concerns about large adverse effects on rare occasions that are unpredictable from observed toxicity data than we do about weak effects on frequent occasions that are predictable from observed toxicity data. The reason is that the ultimate goal is to avoid, despite some uncertainties of information, wrong decisions that would induce considerable impacts of chemical substances on ecosystems and human health.

In practice of risk assessment, such faults may be circumvented by applying the uncertainty factors to the endpoints. However, we evaluated the relative performances of the EC_*x*_ and NOEC with two alternative methods, the frequency-based method and the information-gap method [11]. The former method compares the endpoints as regards the rate or fraction of the estimated endpoints exceeding the maximum acceptable level. The latter method addresses the question of how much uncertainty can be tolerated to meet the required condition that is defined in advance, and chooses a decision that enables us to tolerate the largest uncertainty [11,12,13,14,15]. In the current context, the uncertainty is a value of the within-treatment variance and the decision is whether to evaluate data using the NOEC or using EC_*x*_. The maximum marginal concentration that assures safety of environment is predetermined as TSL (the true safety level) in the present framework, and the required minimum condition is not to exceed TSL. The decision process chooses the method, NOEC or EC_*x*_, that provides an estimate below TSL under a larger value of within-treatment variance, even if that estimate is sometimes higher than that produced by the other method.

Some of the advantages of the EC_*x*_ over the NOEC may depend on features of experimental designs and the stochastic nature of data. The NOEC may be underestimated by weak testing power due to inappropriate designs of experiments, whereas the EC_*x*_ may be biased from the true value in fitting to a particular data set which is subject to stochastic fluctuation [16]. Standard data-fitting procedures, such as the maximum likelihood method and the minimum chi-square estimation, produce best-fit response functions on average, regardless of random data errors. However, deviations of data from the true values result in estimation errors of the benchmark concentration in unpredictable directions as long as it is based on a concentration-response function determined from observed responses.

To quantitatively examine the relative ability of the NOEC and EC_*x*_ to produce robust decisions when there were uncertainties in data, we conducted a Monte Carlo simulation, in which random deviations of data were generated around hypothetical true responses, and the statistical testing and model fitting were carried out.

## Methods

### Outline of the approach

**Fig 1:**
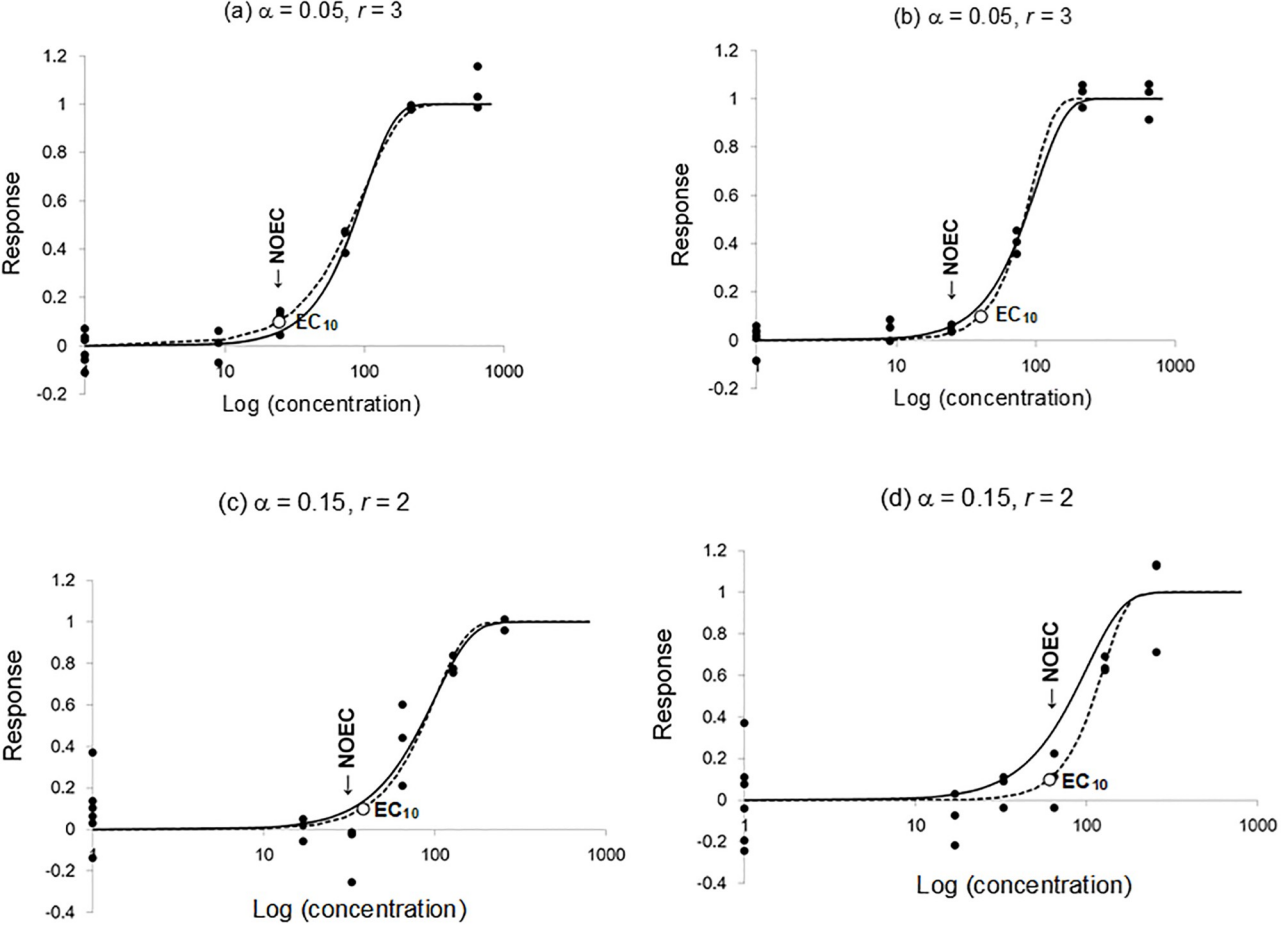
Examples illustrating the relationship between generated response data and concentration-response functions under different levels of uncertainties of data. In all figures (a)—(d), the response slope parameter β of the true concentration-response function (the solid lines) is set to 2 (hence the true EC_10_ is 33), and the responses are continuous. *r* denotes the spacing factor between adjacent test concentrations. For the case of small uncertainties, (a) and (b), the response data (the closed circles) and the concentration-response curve determined from the data (the broken line) do not deviate very much from the true concentration-response function, and estimated EC_10_ and the NOEC are in good accord with the true EC_10_ in many cases although there were still some overestimations of EC_10_ due to uncertainties. For the case of larger uncertainties, (c) and (d), the response data are more likely to deviate from the true concentration-response curve. In the case of (d), the large uncertainties occasionally boosted both the NOEC and EC_10_ beyond the true EC_10_.

A hypothetical true concentration-response function was defined as the asymptotic relationship between toxicant concentrations and adverse responses to the toxicant that would converge if there was no uncertainty in the data (Fig 1). The response was assumed to be either continuous or quantal, and to be measurable in terms of vital properties of individuals or populations, e.g., immobility, mortality, reproductive inhibition, and population growth, whereas we did not hereafter specify a particular kind of responses.

The response *R* was defined in this article as the proportional reduction of the vital rate in the case of a continuous response, whereas for the quantal response, it was defined as the probability of an adverse response occurring among individuals, e.g., death and immobilization. We used the Weibull model for the true response function, $R(x)=1-exp[-{\left(\frac{x}{x_{0}}\right)}^{\beta }]$, in which *x* is the exposure concentration, *x*_0_ is the scale of concentration to which *x* is normalized, and β is the power coefficient that determines the shape of the concentration-response curve. The value of *x*_0_ was set to 100 without loss of generality, and the value of β was set to 2 or 3 for the true model in all simulations. The power coefficient β being 2 or 3 makes the concentration response curve approximately range for one order of magnitude in the toxicant concentration (Fig 1), which is the typical regime of response curves in ecotoxicity experiments.

The benchmark concentration was defined as the concentration at which 10 or 20 percent response was predicted using the true response function without the addition of uncertainty, and was respectively referred to as the TSL_10_ and TSL_20_ hereafter. These values are regarded as the margin of exposures that must not be exceeded and are the strict requirements in our framework, because substantial impacts are supposed to affect the environment or human health when the concentrations are higher than these values. Thus, the TSL_10_ and TSL_20_ were set considerably higher than the conventional benchmark like MATC (the maximum acceptable toxicant concentration), which is equal to the geometric mean of the NOEC and LOEC (the lowest observed effect concentration) and used in the regulatory framework for chemicals (e.g., [17]). The present simulations were designed to evaluate the probability that the NOEC or EC_*x*_ might exceed the TSLs when there are large uncertainties in the data.

### Stochastic generation of hypothetical data

The process of generating data and endowing the simulations with stochasticity differed between the two categories of responses, the continuous and the quantal responses. For the continuous response, stochasticity was assumed to bring about random variations of the performance or the vital rate around the expected values, for both individuals and populations. The expected value of the performance (e.g., fecundity) at exposure concentration *x*, *F*(*x*), was defined as *F*(*x*) = *F*_*c*_(1 − *R*(*x*)), where *F*_*c*_ is the performance without exposure (set to unity without loss of generality), and *R*(*x*) is the fractional response at the exposure concentration *x*. Hypothetical data were generated by random samplings from normal variates with a mean *F*(*x*) and variance α^2^. The magnitude of the uncertainty of the data, here referred to as the scale of the uncertainty, was manipulated by changing the standard deviation α of the random errors. We allowed negative performance values (responses larger than 1) in the random samplings to ensure the unbiased nature of the generated data. The normalization of responses against the mean response in the control was not conducted in our simulations, because the data uncertainty was assumed to occur across replicates within test treatment and then the normalization is not expected to improve estimates of EC_*x*_, whereas it is likely that the normalization for real toxicity data results in biases of EC_*x*_ [18].

For the quantal response, uncertainties were supposed to act to a set of experimental replicates as a whole, and was implemented as an agent generating random perturbation of the probability that a particular quantal response occurs among individuals within a replicate. This treatment of stochasticity postulates that any unidentified microenvironmental effects such as contamination of parasites or pollutants in the test medium, food condition, and handling of test organisms affect the expected response in each experimental replicate such as a test vessel containing 20 neonates of *Daphnia*. The survival probability *S*(*x*), or the probability that the quantal response did not occur for individuals subject to exposure concentration *x*, was defined as *S*(*x*) = *S*_*c*_(1 − *R*(*x*)), where *S*_*c*_ is the survival probability without exposure, and *R*(*x*) is the fractional response among individuals subject to exposure concentration *x*. To be consistent with the case of continuous responses, the survival probability for each experimental replicate was randomly sampled from normal variates with mean *S*(*x*) and variance α^2^. If the sampled value of the survival probability was larger than 1 or smaller than 0, the value was rounded to 1 or 0, respectively, to make the following binomial stochastic process operative. The mortality or the fractional response in each replicate was further generated from the quantal responses at the individual level, and was determined with a binomial stochastic simulation repeated for the number of individuals in each replicate using the above-mentioned survival probability. Therefore, for the quantal response, a purely stochastic process which was not manipulative occurred at the level of individual organisms, and was combined with the uncertainty affecting the survival probability at the level of replicates. The mortality, or the fraction of quantal responses within a replicate, was equated to the fraction of dead or responding individuals among all the individuals in the replicate. The number of individuals per replicates was set 20 in all cases to ensure continuity of the fractions.

The stochastic simulations were repeated for different α values (20 steps), because our major concern was how the distribution of NOEC and EC_*x*_ changed with the uncertainty of the data. We prepared 1000 sets of hypothetical data for each α value with all other features of the model being fixed including the kind of response, the response slope, and the spacing factor between toxicant concentrations (see below).

The experimental design for deriving NOEC and EC_*x*_ from the generated data followed the standard test methods recommended for public documentation [19,20,21,22,23]. We used 6 replicates for the control and 3 replicates for each exposure treatment for both the continuous and quantal responses (the number of individuals in each replicate was assumed 20 as for the quantal response, making the total number of individuals in a treatment or a control to be 60 or 120). The larger number of replicates in the control than in each exposure treatment will increase the power of hypothesis test and the performance of NOEC, but will not have the same effect on EC_*x*_. The number of exposure treatments per test was fixed at 5 in addition to the control, and the spacing factor *r* in the test concentrations was set to 2 or 3 to satisfy the criteria in the standard test methods. We have derived and analyzed the total of 160,000 sets of concentration-response data generated by the simulations for the entire set of parameters and kinds of responses. All simulations were conducted with source codes we made on R [24].

### Statistical analysis

For estimation of the NOEC and EC_*x*_ based on the hypothetical data generated by the simulation, we used the standard statistical methods referenced in the test guidelines [4]. For estimation of EC_*x*_, a particular response model was fit to the data using non-linear least squares to determine the best-fit parameter values for each dataset with the package “nls2” in R [24], which uses the Gauss-Newton or the Golub-Pereyra algorithms. For the quantal response, the raw binary data were converted into a response rate (the number of responding individuals / 20) for each experimental replicate [4]. An alternative more elaborate model-fitting method for quantal response data with replicates within treatment is the maximum likelihood method using the beta-binomial distribution for errors [4]. However, for saving computation time, the same model-fitting procedure was used for both the continuous and the quantal response data in this study.

We also used the Weibull model for fitting dose-response curves to the data, because the use of the same model for both fitting and generating the data was likely to make the model fitting more successful and estimation of EC_*x*_ more precise than any other methods. This operation was expected to favor the commonly accepted remark that EC_*x*_ performed better than NOEC and would be conservative against the alternative conclusions. In cases where the uncertainty scale α was so large that fractions of the entire runs could not be successfully fit to the model because deviations to data did not converge to local minima, we discarded such datasets from the analysis. Inclusion of those data to the analysis considerably worsened the performance of EC_*x*_ in producing safe endpoints under large fluctuations of data (results not shown). However, it may be unrealistic to include those data, because risk assessors are unlikely to practice any non-linear regressions to that kind of data.

To check the model dependency of our results, we examined the performance of the log-logistic and the cumulative log-normal (probit) models in addition to the Weibull model in fitting the data generated with typical parameter settings (β = 2 or 3, and *r* = 2 or 3 for the quantal response). The results were in good accord with our major conclusion (S1 and S2 Figs). We therefore do not discuss the issue of model dependency any further in this article.

For derivation of NOECs, we conducted pairwise comparison tests of the fractional responses between each treatment concentration and the control with Dunnett’s multiple comparison test [4,25]. For the case of quantal response, all response rates were transformed into the arcsine-square root scale in order to stabilize variabilities and to make the distribution closer to the normal. The statistical significance level (type I error rate) for hypothesis testing was 5%. We used two-sided test although the one-sided Dunnett tests and the step-down Jonckheere-Terpstra or other step-down methods are recommended if only one of the monotone trends (responses) is concerned [23]. In our simulations, the true response model was a monotonically increasing function (the Weibull model), implying that increases in responses were the only concern as the adverse effect. However, the data may not be necessarily interpreted in the same manner such that the alternative hypothesis was set as the one-sided responses in all kinds of ecotoxicity tests. Then, the two-sided tests were conducted here in the sake of the testing power to determine NOEC, in order to assure one of our conclusions that NOEC could perform as well as EC_*x*_ in some circumstances to be more conservative.

NOECs were determined in accord with the common definition, the highest test concentration at which responses to the exposure treatment were not statistically significant. There were a few extreme cases in which we were unable to determine a NOEC because all test concentrations resulted in significant responses. We excluded such cases from the analysis, because these occasions were infrequent and limited to the simulations with very small uncertainties, say α = 0.01, and were very unlikely to affect the results.

For evaluating and comparing the robustness between the NOEC and EC_*x*_, we used two alternative methods, one was the frequency of the endpoints that exceeded the TSLs, and the other was the information-gap method [11]. The former is supposed to indicate the probability that an estimated endpoint underestimates the adverse response and makes a false negative result in terms of hazard, and the latter method evaluates risks based on the degree of uncertainties that is permitted for preventing unacceptable consequences by wrong decisions. Ideally, this method is independent of distributions of risk estimates as long as the upper bound of the estimates is not influenced by the distributions, because the risk evaluation with this method focuses on the worst case that is predicted from a particular set of input parameters and magnitudes of uncertainties. Applying this method to the issue in our focus helps determining which endpoint, NOEC or EC_*x*_, is more consistent with the performance that delivered a robust decision of safety in that it does not exceed the TSL, even under high levels of uncertainty.

We regarded the worst case as the least protective value of endpoints, which were set as the 97.5th percentile values of NOEC and EC_*x*_, and were referred to as the *margin of endpoints*. The margin of endpoints increases with the uncertainty scale α, because larger fluctuations of data increases the variance in frequency distributions of estimated endpoints. The largest value of uncertainty scale α (the robustness) at which the margin of endpoints did not marginally exceed the TSL was used as the alternative measure for evaluating the robustness of endpoints, and was denoted α*.

From a set of simulations which produced 1000 values of the same endpoint and the same value of α, the largest 25th value was selected as the margin of endpoint if all simulations are relevant. For the cases where some simulations are irrelevant due to unsuccessful fitting to the model, the value corresponding to the largest 2.5 percent among the available runs of simulations was used as the margin of endpoints. The margins of endpoints were repeatedly estimated for numerous values of α which were systematically changed.

## Results

**Fig 2:**
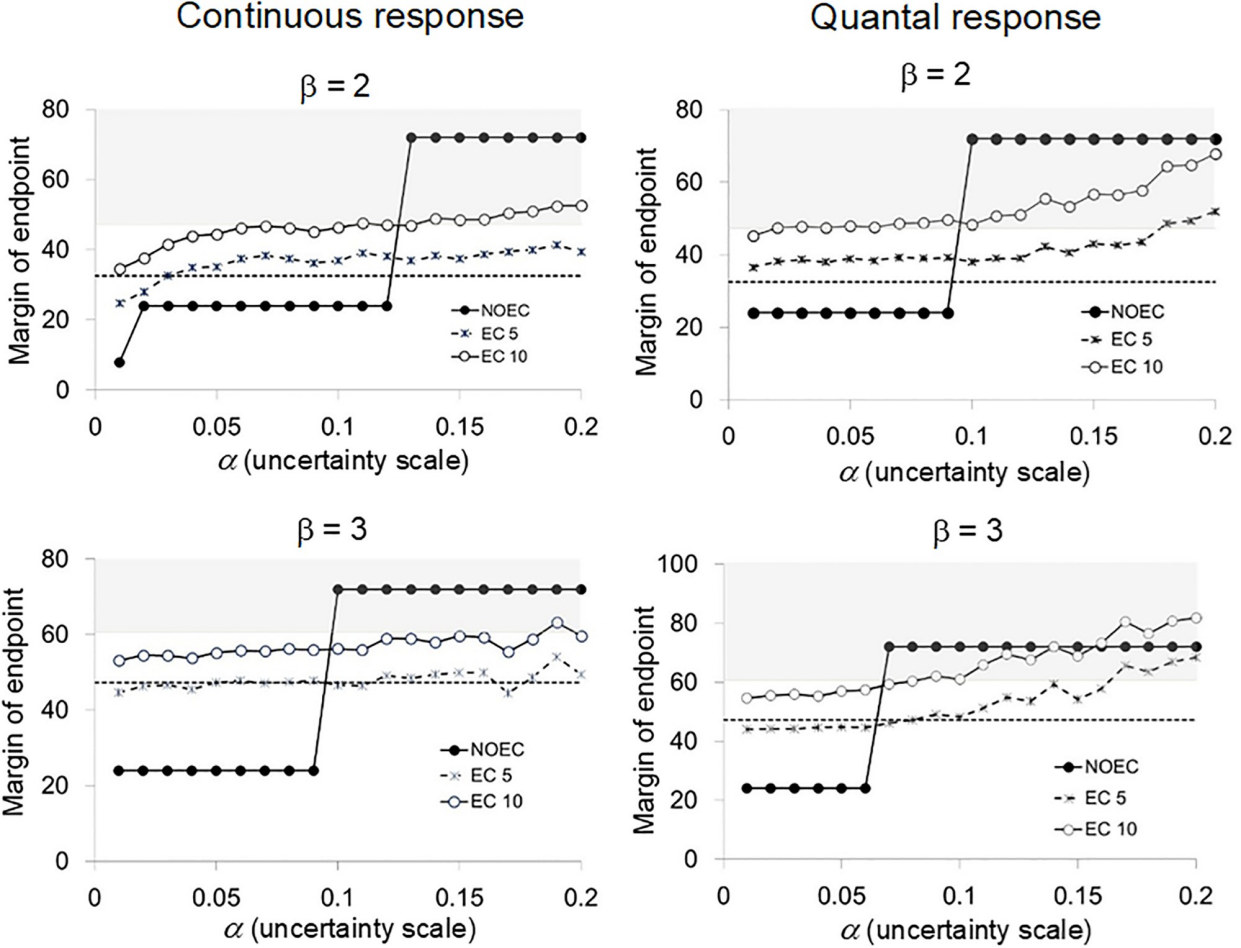
Margins of endpoints (NOEC, EC_5_, and EC_10_) related to the scale of uncertainties (α) for the case where the spacing factor is 3. The shaded area represents the region where the margin of endpoint exceeds the TSL_20_. The broken line denotes the TSL_10_.

**Fig 3:**
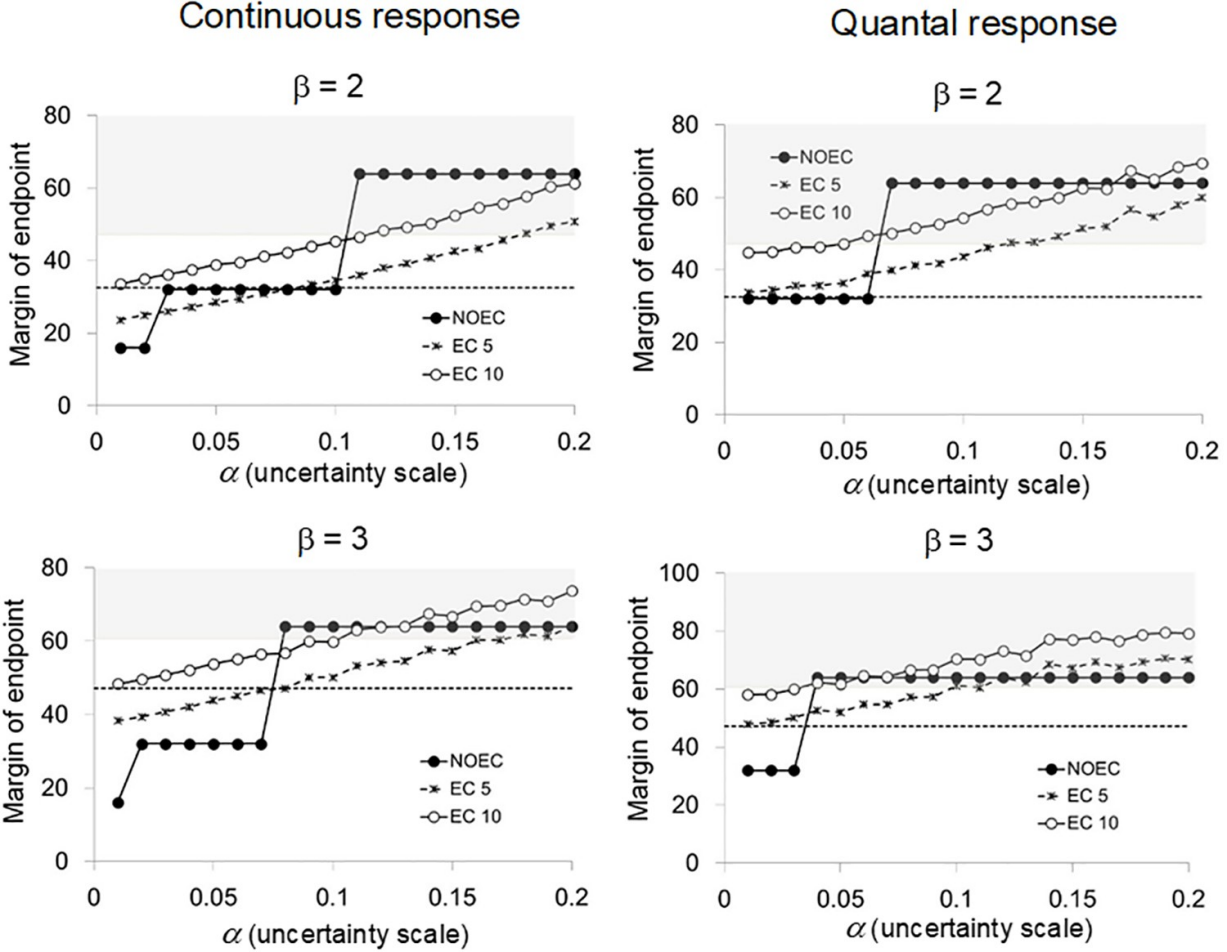
Margins of endpoints (NOEC, EC_5_, and EC_10_) related to the scale of uncertainties (α) for the case where the spacing factor is 2. The shaded area represents the region where the margin of endpoint exceeds the TSL_20_. The broken line denotes the TSL_10_.

We plotted the margins of the endpoints of the NOEC, EC_5_, and EC_10_ based on two response slopes (β = 2 and 3) against the uncertainty scale α in Figs 2 and 3 according to the category of responses and the spacing factor in the test concentrations. The upper bounds of uncertainty α* can be visualized in all figures as the intersections of the margins of endpoint (ordinate) and the base line of the shaded area (for the case of TSL_20_) or the broken line (for the case of TSL_10_). The values of α* are listed in Table 1.

**Table 1:** The robustness for NOEC and EC_5_ or EC_10_.

Continuous response				
	*r* = 3		*r* = 2	
Benchmark	β = 2	β = 3	β = 2	β = 3
NOEC	0.12	0.09	0.1	0.07
EC_5_	0	0.05	0.08	0.08
EC_10_	0.13	0.18	0.11	0.1
Quantal response				
	*r* = 3		*r* = 2	
Benchmark	β = 2	β = 3	β = 2	β = 3
NOEC	0.09	0.06	0.06	0.03
EC_5_	0	0.08	0	0
EC_10_	0.01	0.08	0.05	0.03

The robustness of endpoints was the largest value of uncertainty at which the margins of endpoint did not marginally exceeded the value of TSL_10_ for EC_5_, and TSL_20_ for EC_10_. As for NOEC the values specified at TSL_10_ and TSL_20_ were identical for all cases.

When TSL_20_ was regarded as the benchmark concentration, the EC_5_ always had better performance than the NOEC in all cases (Figs 2 and 3), which implied that the EC_*x*_ specified by an effect rate that is much smaller than the effect rate inducing unacceptable hazards can be an efficient endpoint that is generally more robust than the NOEC in avoiding such hazards. In the following comparisons, the margins of endpoints of EC_5_ were examined with TSL_10_, and those of EC_10_ were examined with TSL_20_. As for the NOEC, the upper bounds of uncertainty were not affected by using TSL_10_ or TSL_20_ with our numerical settings for simulations.

There were no remarkable differences between the continuous and quantal responses with respect to the pattern of changes in the margins of endpoints as the uncertainty scale α changed when the spacing factor *r* was 3 (Fig 2). With the more gradual response slope (β = 2), the margins of both EC_5_ and EC_10_ exceeded the TSL_10_ and TSL_20_ at smaller values of α than the α-value at which the NOEC marginally exceeded these TSLs. On the contrary, for the case where the response slope was steeper (β = 3), the results tended to be reversed: the margins of EC_10_ exceeded the TSL_20_ at larger values of α than the NOEC did for the both kinds of responses. As for EC_5_ it exceeded the TSL_10_ at smaller values of α than the NOEC did for the quantal response, whereas the margins of EC_5_ were very close to the TSL_10_ and the relative performance between the two endpoints was obscure for a wide range of α. The indication here was that when the spacing factor was large (*r* = 3) the relative performance between the NOEC and the EC_5_ or EC_10_ did not have any consistent trends and depends on response slopes of the true model (Table 1).

The relative performance between the NOEC and the EC_*x*_ in making robust decisions subject to uncertainties was not noticeably affected by the spacing factor although the performance of both the NOEC and the EC_*x*_ was lower for the quantal response than for the continuous response when the spacing factor was smaller, *r* = 2 (Fig 3; Table 1). When the benchmark was TSL_10_, the NOEC outperformed or was comparable in performance with the EC_5_ when β = 2 regardless of the spacing factor and the kind of responses, while the NOEC was comparable with the EC_5_ with the steep response slope, β = 3. The EC_10_ clearly outperformed NOEC for keeping the benchmark of TSL_20_ with β = 3 and *r* = 3 regardless of the kinds of response, and slightly performed less than the NOEC with β = 2 for the quantal response. In all other cases, the EC_10_ was comparable with the NOEC. The EC_10_ was not relevant for keeping the benchmark of TSL_10_.

**Fig 4:**
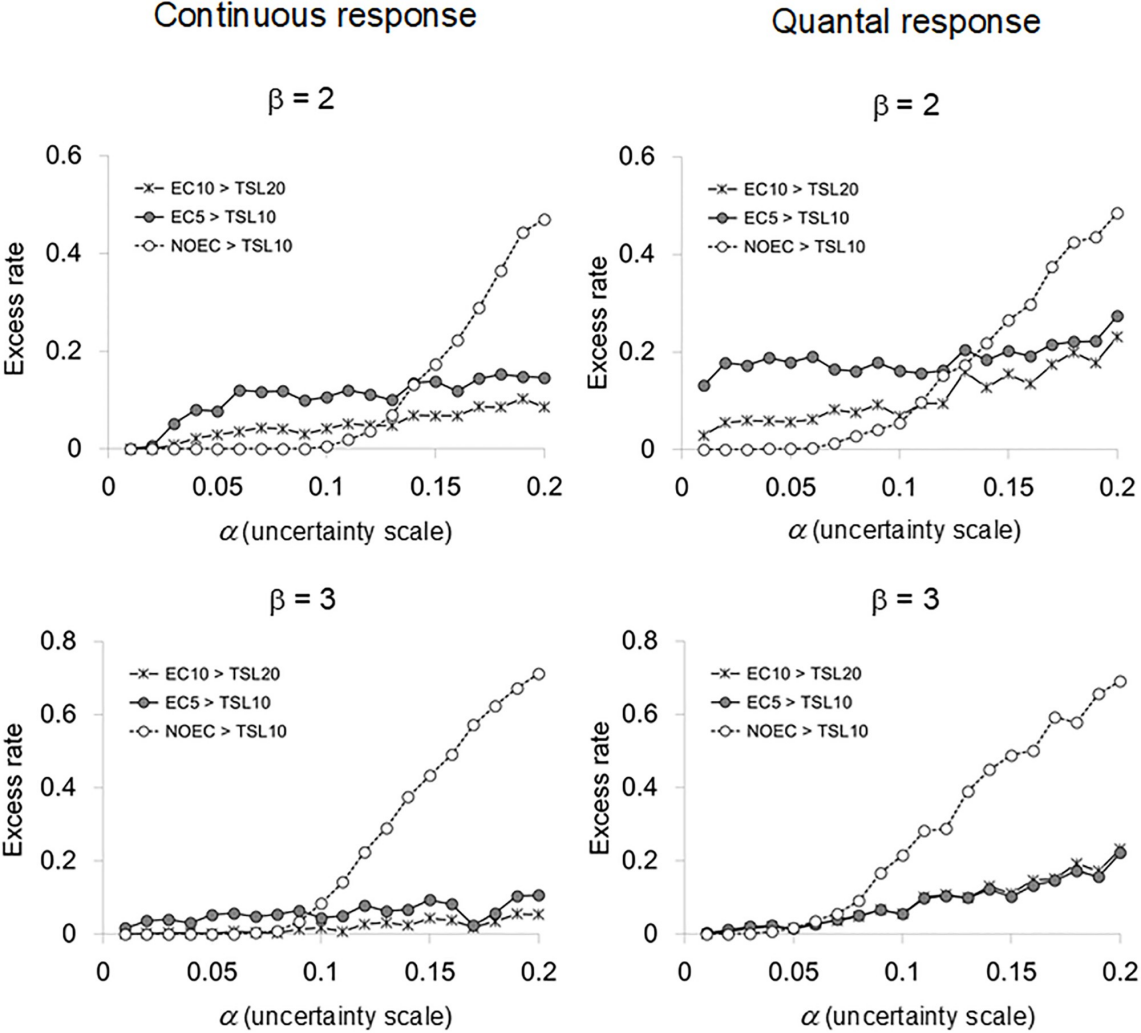
Rates of estimated endpoints (NOEC, EC_5_, and EC_10_) exceeding the predefined benchmarks among relevant simulation runs plotted against the scale of uncertainties when the spacing factor is 3. The comparisons were made between EC_10_ and TSL_20_, between EC_5_ and TSL_10_, and between NOEC and TSL_10_. The results from the last comparisons did not change if TSL_10_ was replaced by TSL_20_ (data not shown).

**Fig 5:**
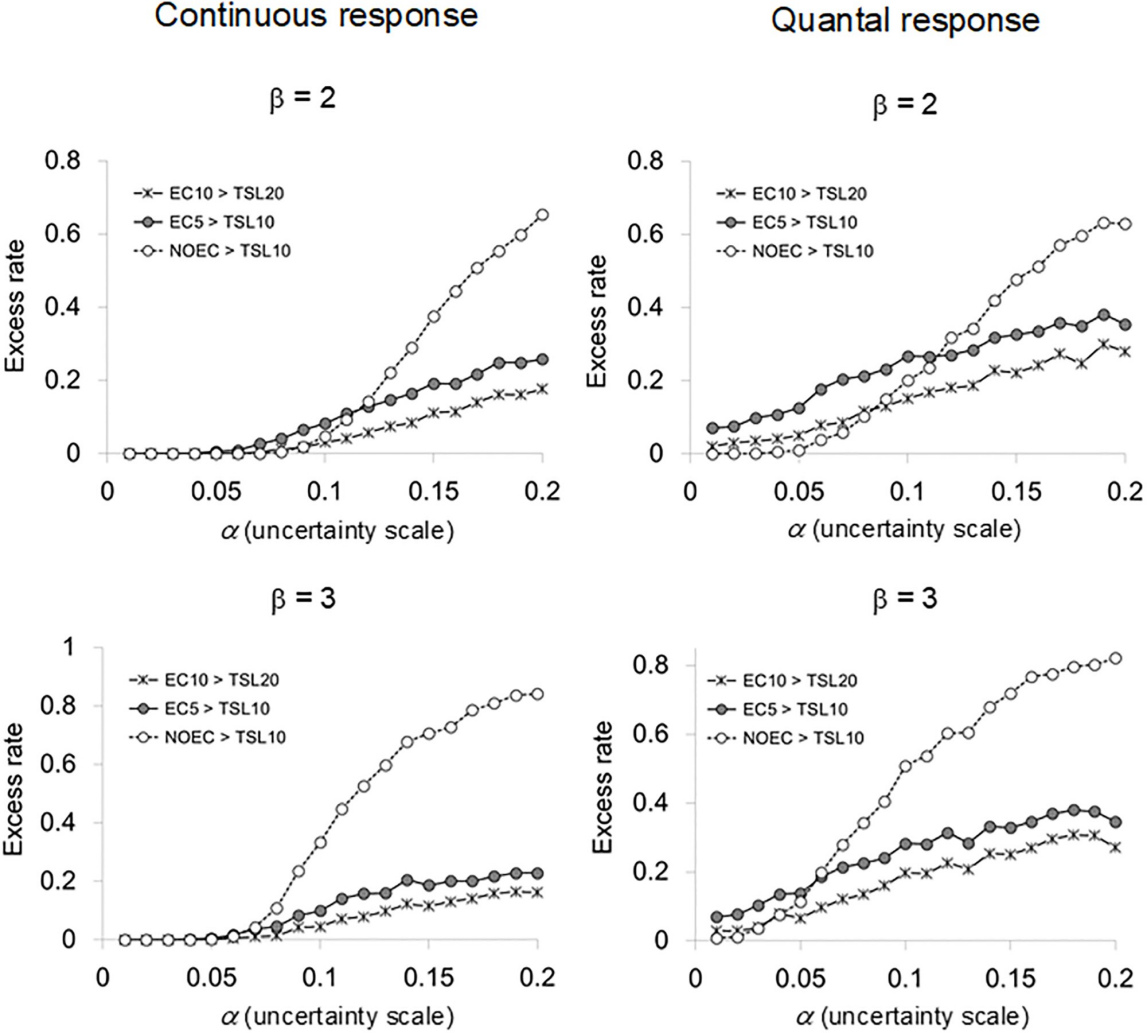
Rates of estimated endpoints (NOEC, EC_5_, and EC_10_) exceeding the predefined benchmarks among relevant simulation runs plotted against the scale of uncertainties when the spacing factor is 2. The comparisons were made between EC_10_ and TSL_20_, between EC_5_ and TSL_10_, and between NOEC and TSL_10_. The results from the last comparisons did not change if TSL_10_ was replaced by TSL_20_ (data not shown).

An alternative method to evaluate the endpoints is based on another feature of distributions of the endpoints (Figs 4 and 5), the frequency or the rate at which each endpoint exceeded the benchmarks for a particular value of uncertainties. These measures inversely indicated the performance of each endpoint as the fraction of excesses (the *excess rate* hereafter), and gave the whole landscape of the performance across different levels of uncertainties.

One of the most common properties with the excess rate, regardless of the spacing factor, the response slope and the kind of response, was that the NOEC had lower excess rates than the EC_*x*_ until the uncertainty scale was large enough for the NOEC to cross over the both benchmarks. Another important observation with the excess rates of the NOEC is that they inflated with the uncertainty scale to much larger values than the excess rates of the EC_*x*_ in all cases (Figs 4 and 5). The reversion of the better performance from the NOEC to the EC_*x*_ occurred with smaller values of α with β = 3 than with β = 2, and with *r* = 3 than with *r* = 2. Thus, the NOEC tended to lose advantage over EC_*x*_ more often (with smaller values of α) when the response shape was steeper, and the exposure concentrations were more spaced in the experiments available for estimating EC_*x*_. The NOEC frequently violated TSLs when uncertainties in data were larger than the upper bound that assured the safety with using the NOEC.

The performance of EC_*x*_ in terms of the excess rate was drastically changed by the alternative assumption of response slopes for the case of *r* = 3 (Fig 4), whereas it was fairly independent of the response slope for the case of *r* = 2 (Fig 5). The considerably lower performance of EC_*x*_ with the gradual response slope (β = 2) in comparison to the case with the steep response slope (β = 3) was also observed in the upper bounds of uncertainties (Fig 2). For the case of *r* = 3 and β = 2, the excess rate was larger than 10 percent even when the uncertainty scale was very small (larger than 0.05 for the continuous response, and equal to or larger than 0.01 for the quantal response). The larger improvement of the excess rate for the case of *r* = 3 and β = 3 might be a consequence of the treatment that the datasets in which the non-linear regression resulted in unsuccessful fitting to the model were excluded from the analysis (see “Statistical Analysis”).

In summary, the entire simulation suggested that, based on the criterion that an endpoint was prioritized if it assured the safety defined by the unacceptable level of hazards even with the larger uncertainties, the EC_*x*_ clearly outperformed the NOEC in two situations: one used the EC_5_ as the endpoint for avoiding the benchmark of TSL_20_, and the other assumed that the response was continuous and steep, β = 3, and used the EC_10_ as the endpoint for avoiding the benchmark of TSL_20_. In the rest of all cases examined, the performance was comparable between the two kinds of endpoints, and any consistent trends were not observed as regards the relative performance between the NOEC and the EC_*x*_.

The alternative interpretation of data, which focused on the rate at which estimated endpoints exceeded the benchmarks, has suggested that the NOEC tended to outperform the EC_*x*_ only if the slope of the true response function was gradual and the uncertainty scale was less than 10 percent, or the slope of the true response function was steep and the uncertainty scale was less than 5 percent. Generally, the excess rates of the NOEC were inflated to much higher values than those of the EC_*x*_ with larger uncertainties in data.

## Discussion

One of the most substantial criticisms of the NOEC and NOAEL (no observed adverse effect level) as endpoints in toxicology is mostly based on the process of statistical hypothesis testing, which is prone to type II error, the incorrect conclusion that there is no effect (or no difference from the control) when there is an effect [1]. Recent development of test guidelines has come to include the validity requirement of the hypothesis testing which is not less than 75 percent testing power (less than 25 percent type II error) [23], although the type II error is still under less strict control than for the type I error and could be large if the sample size is small and the data uncertainty is large. Unlike heuristic scientific researches in which the reliability of positive results is prioritized to the certainty of negative results, type II errors should be of greater concern than type I errors in regulatory sciences or the regulation of chemicals, because the decisions for protection of the environment must be biased, if it is, toward safety rather than certainty of positive results.

In the model-based approach, one of the best models is determined by estimating model parameters with the maximum likelihood or other model-fitting procedures, and the endpoints, such as the EC_*x*_, are determined by extrapolation or interpolation of the model. Thus, the endpoints derived from the model-fitting procedure may be apparently less biased and more independent of type II errors than the endpoints derived from the hypothesis-testing procedure if the EC_*x*_ is documented with acceptably narrow confidence bounds. The above claim is in accord with the present results, which indicated that the NOEC tended to exceed the benchmark concentration at higher rates than the EC_*x*_ if uncertainties of data were large.

The type II error is not defined with respect to the EC_*x*_ because the estimation of the EC_*x*_ is not based on hypothesis testing. However, our simulations have suggested that estimation errors of responses may cause appearance of type II errors even with the model-based decisions in the sense that estimates of EC_*x*_ may be incidentally judged above the benchmark concentration, and the risk assessment might cause a falsely negative decision. Such risks may be prevented by fine documentation on the confidence bounds of EC_*x*_ which is recommended by a test guideline recently rectified [23]. The vulnerability to the apparent and the true type II errors may be considerably larger with the EC_*x*_ than with the NOEC if the fluctuations of data are well within 10 percent coefficient of variation, and vice versa.

The other criticism of the NOEC and related concepts is based on the argument that the NOEC is not an unbiased statistic with respect to testing power. However, one of the most important purposes of risk assessment at the lowest tier (primary screening) is to determine the critical environmental exposure concentration that ensures the absence of unacceptable hazards to the environment or to human health, even taking the large uncertainties in the toxicological data into account, rather than to obtain unbiased estimates of responses to toxicants. In addition, it is usually impossible to quantify the uncertainties or to estimate the probability distribution of endpoints until toxicity tests for the same toxicant have been repeated many times under the same test conditions.

One of the relevant conceptual bases for decision making with data characterized by such unknown uncertainties is the information-gap method, which was developed in operations research and has been applied to environmental sciences [11,12,13]. This method facilitates selection of the best management scenario among all options in a way that takes account of the largest uncertainties. The selection is subject to the condition that a minimum requirement be satisfied, even for the worst case in the selected scenario. The scenario that permits the largest uncertainty under this condition is selected. The information-gap method was thus created to ensure that the most robust decision would be made, i.e., that the minimum requirement would be met even with the largest uncertainty [11].

The information-gap methodology may be facilitated for evaluating toxicological endpoints by the fact that toxicological data available for regulation are often very restrictive as regards the number of experimental treatments for different exposure concentrations and the number of replicates within treatments. In a worst-case scenario, these tests may result in a long-tailed distribution of the estimated endpoints or inflated estimates of the endpoint, because such estimates are sensitive to limited sample sizes. Analogous phenomena have also been pointed out in the application of model-based approaches to the distribution of species sensitivity [26].

It would be one of the relevant directions for addressing this issue to evaluate the uncertainty in data and take the uncertainty into account in the estimation of model parameters from the dataset by means of bootstrapping, jackknife resampling, or other resampling methods for bias reduction [25, 27, 28]. Such resampling methods provide estimation error distributions and conduct the bias reduction from sampled data, however, cannot reconstruct uncertainty that is not reflected in the derived dataset.

The other point is related to the procedure that has been developed to ensure the safety in risk assessment. Such procedures have been traditionally implemented by applying uncertainty factors (UF) to experimentally determined endpoints [29]. However, we seldom know the appropriate value of UF that can safely prevent the very rare occasions that a set of experimental data happens to underestimate the true response rate that would result in real hazards to the environment. The information-gap methodology may lead to a solution which minimizes the incidental occurrence of the unacceptable hazards.

The results of the present simulations have implied that the NOEC and the EC_*x*_ are comparable in the reliability and the robustness as endpoints in limited situations: the relative ability between the two types of endpoints depends on the magnitude of uncertainties in data. The largest uncertainties of data that did not allow the margin of endpoints to exceed the maximum acceptable concentration were generally comparable between the NOEC and the EC_*x*_, regardless of the response slopes and experimental designs, as long as EC_10_ and TSL_20_ were used for the comparison. The EC_*x*_ did not clearly outperform the NOEC unless the EC_5_ was used as the endpoint. However, the results that the margins of EC_*x*_ were considerably larger than the margins of NOEC in many cases where the uncertainties in data were small indicates that the NOEC produces more robust and protective endpoints than EC_*x*_ if the uncertainty in data is well regulated. The margin of NOEC sharply crossed the margin of EC_*x*_ in most cases as the uncertainty scale increased. With uncertainties in data larger than certain values, the margin of NOEC inflated beyond that of EC_*x*_, due to rapid reduction of the testing power. The critical values of uncertainties with which the margins of NOEC and EC_*x*_ reverse in their magnitudes were around 10 percent, which varied from 5 to 12 percent depending on the kind of responses and the response slopes.

The above trend was consistent with the observed rates at which the endpoints exceeded the TSLs. With small uncertainties in data, the NOEC did not exceed the TSLs for the most combinations of parameters, whereas with larger uncertainties it exceeded TSLs at much higher rates than the rate at which the EC_*x*_ exceeded TSLs.

The overall conclusion of this study is that provided that the magnitude of uncertainties is well less than 10 percent of coefficient of variation, the NOEC can be comparable with or even outperform the EC_*x*_ in producing robust benchmarks since they can keep safety of toxicant concentrations. However, with larger uncertainties in data, EC_*x*_ is likely to outperform the NOEC. The EC_*x*_ is much more likely than the NOEC to indicate the upper limit of toxicant concentrations not exceeding the unacceptable level. The critical value of uncertainties at which the NOEC inflates and loses its advantage over the EC_*x*_ would be between 5 and 10 percent of coefficient of variations in responses. This observation may make the supposed advantage of the EC_*x*_ over the NOEC more ambiguous, because some ecotoxicity test guidelines demand the coefficient of variation of observed values in the control to be less than certain percentages (10 percent for the algae growth test [20] and 25 percent for the *Daphnia* reproduction test [21]).

It should be noted, however, that there is an important another fault with the NOEC from a regulatory standpoint. If the data are derived from an experiment in which there are extremely high repeatabilities across replicates, NOECs are often decided as being equal to the lowest test concentration, which predicts only a negligible response, implying that the effect size is too small to induce any significant risks [25]. This practice may lead to overestimation of risks for some substances when they are examined with accurate experiments entailing overly strong testing power.

Because a NOEC is not based on quantitative evaluation of toxicants’ effects, a NOEC may be at best as relevant as the EC_*x*_ in the primary screening or first tier risk assessment, in which benchmark concentrations are determined to ensure safety. The higher tier of risk assessment, in which quantitative evaluations of risk are required, may be more appropriately based on concentration-response relationships. How to incorporate these two different approaches, the model-based method and the statistical hypothesis-testing method, in an integrated framework of risk assessment and regulation remains a future task of toxicology.

## Supporting information

S1 Fig Results of the simulation using the cumulative log-normal model as the true concentration-response function. (TIF)

S2 Fig Results of the simulation using the log-logistic model as the true concentration-response function. (TIF)

S1 File Statistics of continuous response data. (XLSX)

S2 File Statistics of discrete response data. (XLSX)
