# Supplementary figures and images for: Relative robustness of NOEC and ECx against large uncertainties in data
Source: PLoS One. 2018 Nov 28;13(11):e0206901. doi: 10.1371/journal.pone.0206901 (PMC6261558; doi:10.1371/journal.pone.0206901)

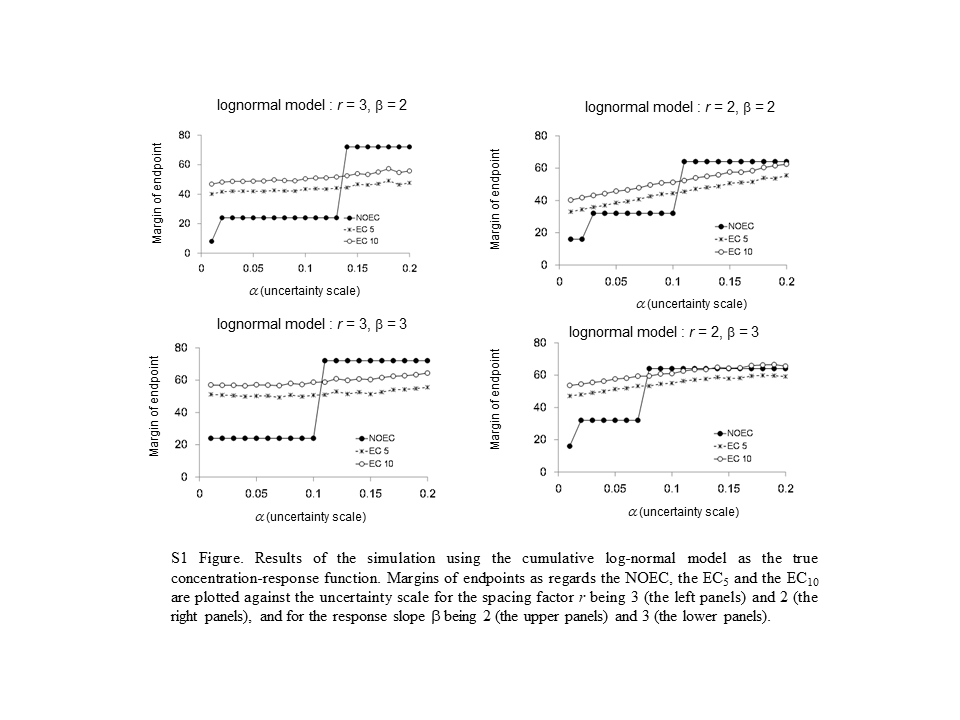

Supplement: S1 Fig — (TIF) [file pone.0206901.s001.tif]

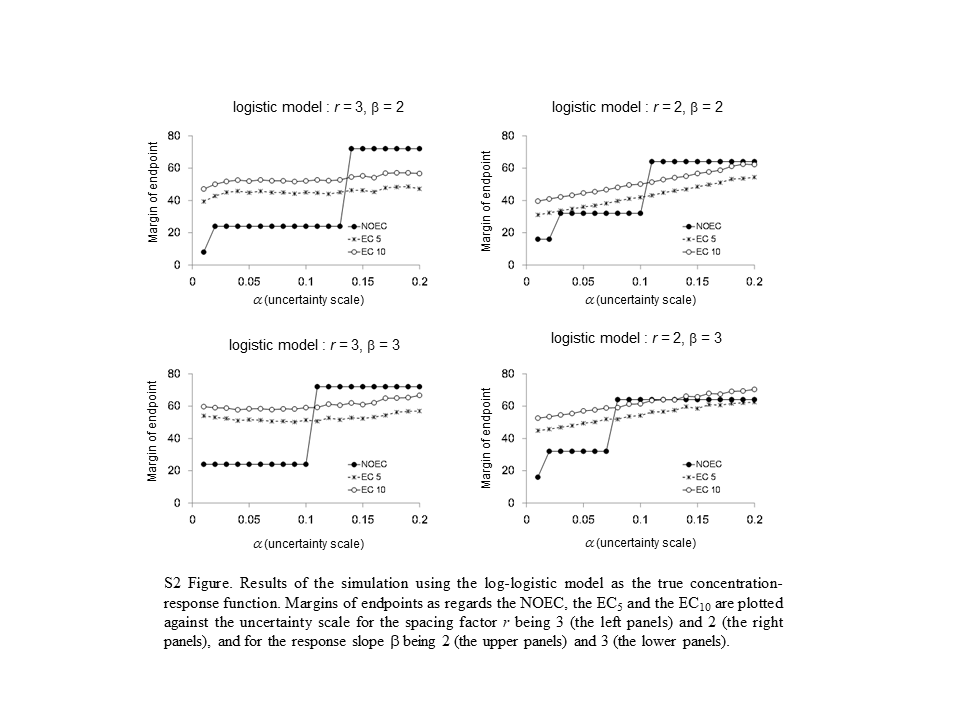

Supplement: S2 Fig — (TIF) [file pone.0206901.s002.tif]
